# Utility of anterior wall of greater trochanter in predicting femoral anteversion angle: a three-dimensional computed tomography-based simulation study

**DOI:** 10.1186/s13018-022-03313-z

**Published:** 2022-09-10

**Authors:** Masahiro Suzuki, Koichi Kinoshita, Tetsuya Sakamoto, Hajime Seo, Sakae Kinoshita, Ichiro Yoshimura, Takuaki Yamamoto

**Affiliations:** Department of Orthopedic Surgery, Fukuoka University Faculty of Medicine, 7-45-1 Nanakuma, Jonan-ku, Fukuoka, 814-0180 Japan

**Keywords:** Femoral anteversion angle, Anterior wall angle, Hip surgery, Anatomical landmark

## Abstract

**Background:**

The femoral anteversion angle is an important factor in performing surgery in the proximal part of the femur. Predicting the femoral anteversion angle based on the morphology of the proximal femur is clinically useful. The purpose of this study was to investigate whether an anatomical landmark can be used to predict the femoral anteversion angle intraoperatively.

**Materials and methods:**

We analysed CT data obtained from 100 hips in 69 patients with osteonecrosis of the femoral head with no more than 2 mm collapse and no evidence of osteoarthritic changes. The measured variables were the femoral anteversion angle, the femoral neck-shaft angle, and the AW angle (defined as the angle between the femoral shaft axis and the tangential line of the anterior wall of the greater trochanter). The correlations between variables were also investigated. Multiple regression analysis by the forced input method was performed for the degree of femoral anteversion angle, using sex and the AW angle as explanatory variables.

**Results:**

On CT, the mean femoral anteversion angle was 14.8° ± 10.8°, the mean AW angle was 17.5° ± 8.0°, and the mean femoral neck-shaft angle was 127.3° ± 5.4°. There was a positive correlation between the femoral anteversion angle and the AW angle. The approximation equations based on the multiple regression analysis were as follows: male femoral anteversion angle = AW angle × 0.7 − 0.7 and female femoral anteversion angle = AW angle × 0.7 + 4.3.

**Conclusions:**

Femoral anteversion angle can be predicted based on the AW angle of the greater trochanter.

## Introduction

The femoral neck-shaft angle and femoral anteversion angle are important factors in performing surgery in the proximal part of the femur. These angles need to be considered when using lag screws and pins to treat femoral neck fracture and slipped capital femoral epiphysis, and when performing core decompression for osteonecrosis [1–3]. In addition, femoral anteversion is an important factor in obtaining an appropriate postoperative intact area in transtrochanteric rotational osteotomy for osteonecrosis, Perthes disease, and severe cases of slipped capital femoral epiphysis [4, 5].

Femoral anteversion angle has been measured based on several methods, including fluoroscopy, radiography, ultrasound, magnetic resonance imaging (MRI), and computed tomography (CT) [6–11]. Among them, CT of the whole femur (from the femoral head to the distal femoral condyles) has been reported to be the most accurate method for the measurement of femoral anteversion angle [10–12]. However, CT of the whole femur is not always possible for all cases because of patient age, pregnancy, radiation exposure, and hospital economy and ability [13–15]. In such cases, femoral anteversion angle has generally been measured using plain radiographs, which is also known to be influenced by the leg position when taking radiographs [16]. It is often difficult to obtain a precise and appropriate leg position in patients with hip pain and restricted range of motion. Therefore, the intraoperative use of an intensifier has been widely adopted to reconfirm the femoral anteversion angle at the time of surgery, but the duration of use should be as short as possible.

In total knee arthroplasty, several anatomical landmarks have been proposed as the predictor to adjust the rotational alignment of the femoral and tibial components, including the anteroposterior axis of the femur (Whiteside’s line) [17] and the anteroposterior axis of the tibia (Akagi’s line) [18]. The utility of these landmarks has been supported by several studies [19–21]. Similarly, in the hip joint, predicting femoral anteversion angle based on the morphology of the proximal femur is clinically useful [22–25]. The posterior lesser trochanter line has been reported as an intraoperative reference guide to predict the anteversion angle [24, 25], but it cannot be applied when using the anterior approach and its validity is still controversial [26].

To identify a useful anatomical landmark to predict the femoral anteversion angle intraoperatively, we focussed on the anterior wall angle (AW angle) of the greater trochanter (GT), which was defined as the angle between the femoral shaft axis and the tangential line of the anterior wall of the GT. We hypothesized that the AW angle is an anatomical landmark for predicting femoral anteversion angle.

## Methods

### Patients

This study was approved by our institutional review board (approval number: U20-12-001). We retrospectively reviewed CT data from 148 hips of 74 consecutive patients diagnosed with osteonecrosis of the femoral head (ONFH) in our hospital from April 2015 to February 2020. The diagnosis of ONFH was based on previously reported criteria [27]. CT examination of both hips was performed for all patients, even if no osteonecrosis was present on the contralateral side. To avoid any secondary changes in the morphology of the femoral head and GT, 48 of the 148 hips were excluded because there was more than 2 mm collapse (12 hips), evidence of osteoarthritic changes (24 hips), or a history of trauma or previous hip surgery (12 hips).

The final study cohort included 100 hips of 69 patients (Table 1). The patients comprised 34 males (53 hips) and 35 females (47 hips). Their mean age at the time of CT was 53.9 years. Their mean height was 162.2 cm. There were 33 normal hips, 3 hips classified as stage 1 ONFH, 21 hips classified as stage 2 ONFH, and 43 hips classified as stage 3A ONFH (less than 2 mm collapse) [28].

**Table 1:** Patients’ characteristics

	*n* = 100 (69 patients)
Age when CT was performed (years)	53.9 ± 17.3 (15–88)
Sex: male/female	53 (34 patients)/47 (35 patients)
Height (cm)	162.2 ± 9.0 (142.5–182.0)

Age and height data are expressed as the mean ± standard deviation (range); sex data are expressed as the number of hips (number of patients)

### CT imaging

All CT imaging was carried out using the Aquilion TSX-101A/HA (Toshiba Medical Systems, Tochigi, Japan) with the patient in the supine position and symmetrically placed in the scanner as shown by the scout views. The images were obtained at 2-mm intervals from the anterosuperior iliac spine to the knee, including the entirety of the distal femoral condyles. After downloading the CT data in Digital Imaging and Communications in Medicine format (National Electrical Manufacturers Association, Rosslyn, VA, USA) onto a personal computer, multiplanar reconstructed images were obtained using CT-based simulation software (ZedOsteotomy; LEXI, Tokyo, Japan).

### Definition of level of anterior wall of the GT

**Fig. 1:**
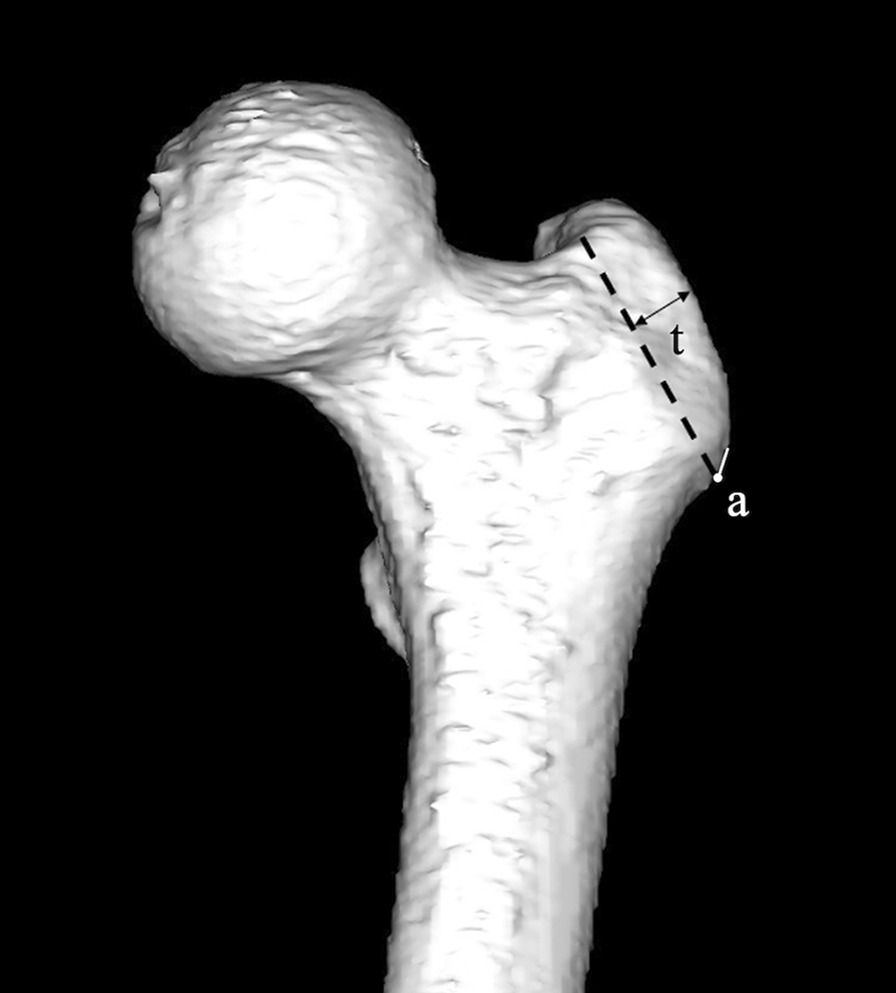
The level of the anterior wall of the greater trochanter (dotted line) on anteroposterior view using the International Society of Biomechanics coordinate system. The simulated cut of the greater trochanter passes through the point 5 mm distal to the lateral ridge of the greater trochanter (a), giving a maximum thickness (t) of 10 mm. The cutting line was set based on the osteotomy line of the GT commonly used in hip joint-preserving osteotomies such as transtrochanteric rotational osteotomy and transposition osteotomy of the acetabulum [29, 30]

**Fig. 2:**
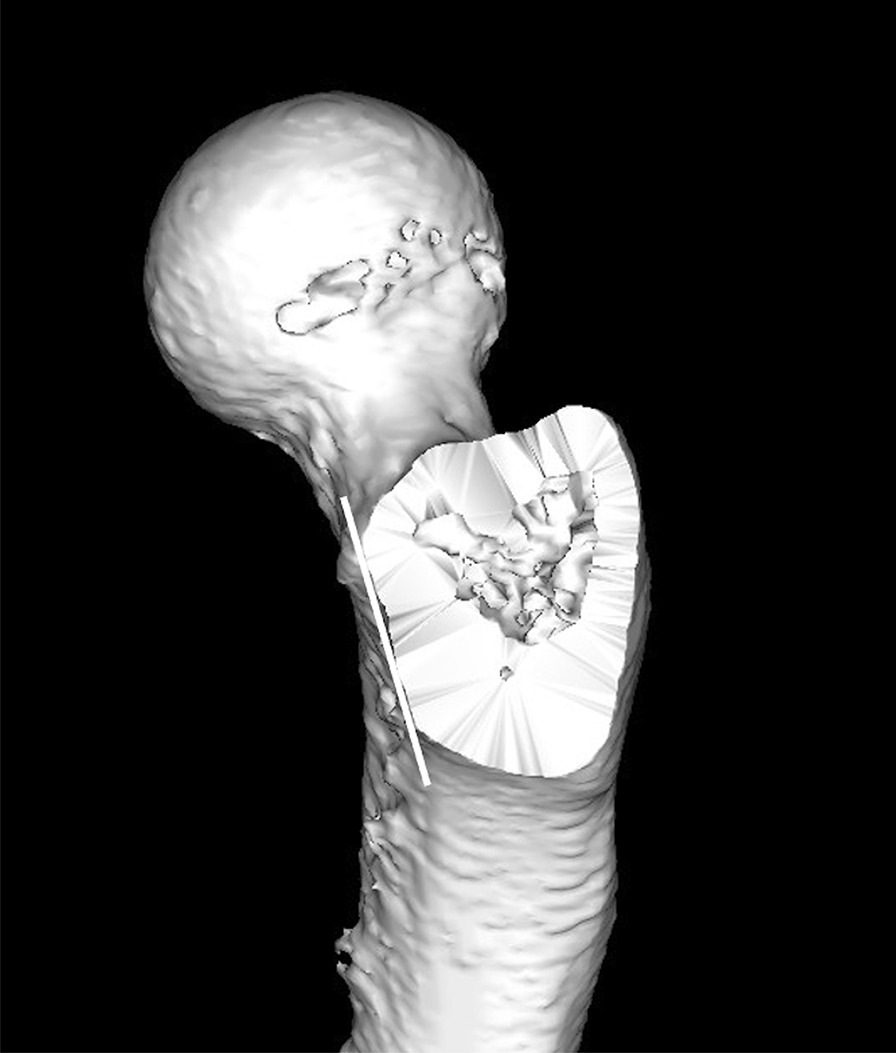
The plane of osteotomy site of the greater trochanter. The line tangential to the anterior wall was defined as the anterior wall line

We simulated the cutting of the GT passing through a point 5 mm distal to the lateral ridge of the GT, giving a maximum thickness of 10 mm (Fig. 1). The anterior wall of the cut surface of the GT was then nearly flat in all cases. The line tangential to the anterior wall was defined as the anterior wall line (Fig. 2).

### Definition of variables

**Fig. 3:**
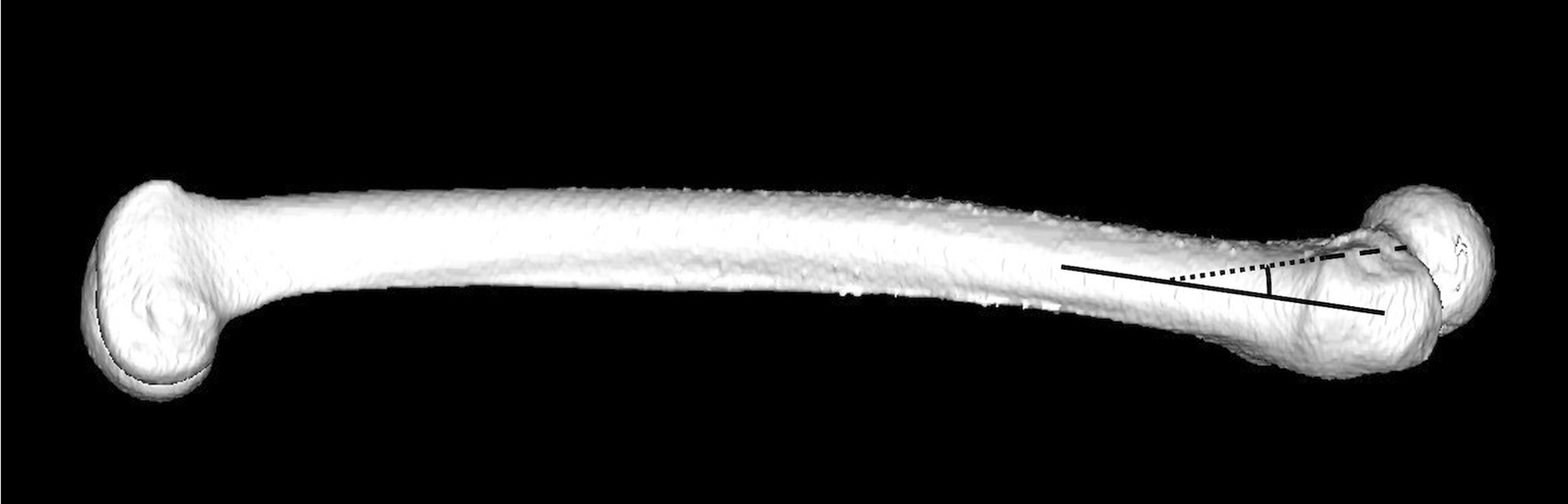
The anterior wall angle on sagittal view using the International Society of Biomechanics coordinate system. The anterior wall angle (AW angle) is defined as the angle between the femoral shaft axis (solid line) and the anterior wall line (dotted line)

The centre of the femoral head was determined based on the sphere that best-fit the surface of the femoral head. The femoral neck axis was defined three-dimensionally as a line passing through the centre of the femoral head and the midpoint of the narrowest part of the femoral neck. The femoral shaft axis was determined as a line connecting the centre of the medullary canal in a transverse section at the base of the lesser trochanter and in a transverse section 5 cm further distally. Femoral anteversion angle was defined as the angle between the femoral neck axis and a line connecting the posterior aspect of the medial and lateral femoral condyles (posterior condylar line) on the axial view [31]. The femoral neck-shaft angle was defined as the angle between the femoral shaft axis and the femoral neck axis on the anteroposterior view on the tabletop coordinate system [31]. The AW angle was defined as the angle between the femoral shaft axis and the anterior wall line on the sagittal view using the International Society of Biomechanics coordinate system [32] (Fig. 3).

### Statistical analysis

CT images were measured independently by two orthopaedic surgeons (MS, SK). In addition, the same observer reviewed the radiographs twice on different days, and the average values were calculated. The intraobserver and interobserver reliabilities were assessed using interclass correlation coefficients. The relationships between the femoral anteversion angle, AW angle, and femoral neck-shaft angle were assessed using Pearson’s correlation coefficient. The chi-square test was used to compare categorical data, such as sex. In addition, multiple regression analysis by the forced input method was performed to assess the effect on the degree of femoral anteversion angle; the explanatory variables were sex and variables that showed a strong correlation with femoral anteversion angle. Sex was included as an explanatory variable because several previous studies have shown that females have significantly greater femoral anteversion angle than males [33, 34]. Before performing the multiple regression analysis, the normality of the variables was confirmed by the Shapiro–Wilk test, and the shape of the distribution was confirmed by a histogram. SPSS version 20.0 (IBM Corp., Armonk, NY, USA) was used for the statistical analysis. A *P* value of < 0.05 was considered statistically significant.

## Results

**Fig. 4:**
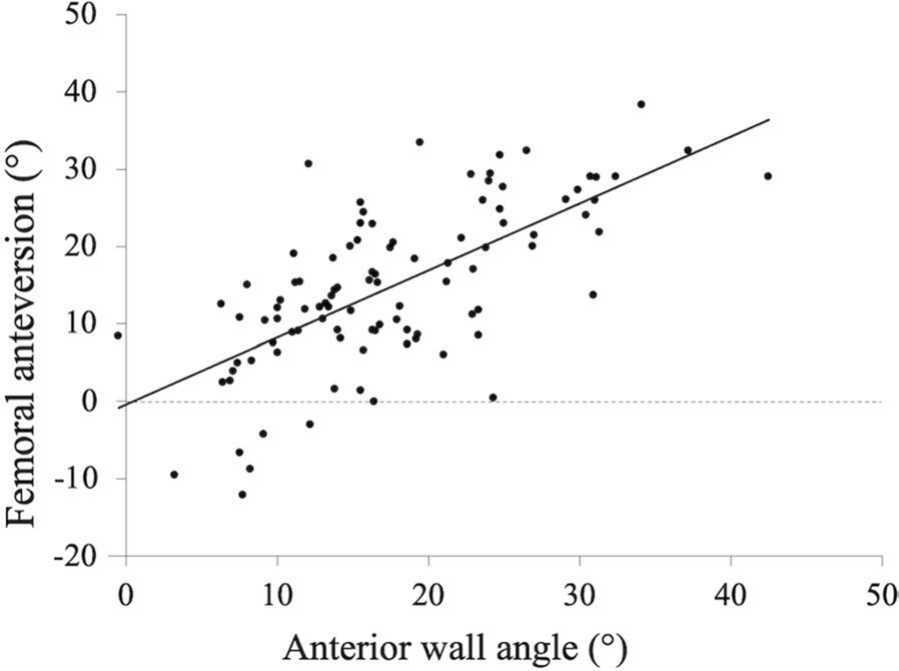
Graph showing the relationship between the femoral anteversion and anterior wall angle

The mean ± standard deviation femoral anteversion angle was 14.8° ± 10.8° (range, − 12.1–38.4°), the mean AW angle was 17.5° ± 8.0° (range, − 0.5–42.5°), and the mean femoral neck-shaft angle was 127.3° ± 5.4° (range, 116.6–143.2°). All measurements (femoral anteversion angle, AW angle, and femoral neck-shaft angle) showed good intraobserver reliability (0.99, 0.96, and 0.99, respectively) and interobserver reliability (0.98, 0.94, and 0.97, respectively). Six patients (5 males, 1 female) had a femoral anteversion angle of < 0° (femoral retroversion). The femoral anteversion angle was positively correlated with the AW angle (*P* < 0.001, *r* = 0.67) (Fig. 4) but was not correlated with the femoral neck-shaft angle (*P* = 0.59, *r* = − 0.05). The mean femoral anteversion angle and AW angle were significantly greater in females than in males (Table 2). There was no significant difference between ONFH and normal hips in the femoral anteversion angle, AW angle, and femoral neck-shaft angle (Table 3).

**Table 2:** Sex differences in the femoral anteversion angle, anterior wall angle, and femoral neck-shaft angle

Parameter	Males (53 hips)	Females (47 hips)	*P* value
Femoral anteversion angle (°)	10.2 ± 8.7 (− 12.1–33.4)	19.8 ± 9.6 (− 8.8–38.4)	< 0.001
Anterior wall angle (°)	14.6 ± 6.6 (− 0.5–31.3)	20.8 ± 8.2 (8.0–42.5)	< 0.001
Femoral neck-shaft angle (°)	127.3 ± 5.2 (116.6–141.0)	127.3 ± 5.7 (116.6–143.2)	0.95

All data are expressed as the mean ± standard deviation (range)

**Table 3:** Comparison in the femoral anteversion angle, anterior wall angle, and femoral neck-shaft angle between osteonecrosis of femoral head (ONFH) and normal hips

Parameter	ONFH (67 hips)	Normal (33 hips)	*P* value
Femoral anteversion angle (°)	14.7 ± 10.5 (− 12.1–38.4)	15.0 ± 10.0 (− 8.8–38.4)	0.899
Anterior wall angle (°)	17.9 ± 8.1 (− 0.5–37.2)	16.9 ± 8.0 (7.5–42.5)	0.410
Femoral neck-shaft angle (°)	127.6 ± 5.5 (116.6–143.2)	126.7 ± 5.2 (116.6–139.9)	0.402

All data are expressed as the mean ± standard deviation (range)

No variables significantly deviated from the normal distribution or had a biased frequency. Therefore, dummy variable conversion and change of variables were not performed. Multiple regression analysis was performed to predict the femoral anteversion angle based on the AW angle and sex as explanatory variables. The result of the multiple regression analysis for femoral anteversion angle is shown in Table 4. A significant regression equation was found [*F* (2, 97) = 49.945, *P* < 0.000] with an R^2^ value of 0.507. The predicted femoral anteversion angle was equal to 4.330 + 0.744 (AW angle) − 4.998 (sex), when sex was coded as 0 = female and 1 = male. Therefore, the approximation equations for each sex were as follows:

$${\text{Male}}\;{\text{femoral}}\;{\text{anteversion}}\;{\text{angle}} = {\text{AW - angle}} \times 0.7 - 0.7$$

$${\text{Female}}\;{\text{femoral}}\;{\text{anteversion}}\;{\text{angle}} = {\text{AW - angle}} \times 0.7 + 4.3$$

The femoral anteversion angle increased by 0.7° with each degree increase in the AW angle, and the femoral anteversion angle was about 5° greater in females than males, even at the same AW angle. Both the AW angle and sex were significant predictors of femoral anteversion angle. The result of the analysis of variance was statistically significant. The Durbin–Watson ratio was 1.789, which indicated only a very mild amount of autocorrelation, and there were no outliers whose predicted values exceeded ± 3 standard deviations with respect to the measured values. The error between the result of regression equation and femoral anteversion angle was more than 10° in 17% (17 of 100 hips), which included 6 cases with femoral retroversion.

**Table 4:** Results of multiple regression analysis for femoral anteversion angle

Variable	*B*	*SE B*	*β*	*P* value	95% confidence interval, CI	
					Lower limit	Upper limit
Constant	4.330	2.312		0.064	− 0.258	8.918
Anterior wall angle	0.744	1.586	0.581	0.002	0.547	0.941
Sex	− 4.998	0.099	− 0.244	0.000	− 8.145	− 1.852

Adjusted *R*^2^ = 0.507, analysis of variance *P* < 0.001

## Discussion

This simulation study demonstrated that the femoral anteversion angle can be predicted based on the AW angle of the GT. Our findings indicate that the anterior wall of the GT may be used to predict the degree of femoral anteversion angle intraoperatively, even if CT of the whole femur (from the femoral head to the distal femoral condyles) cannot be performed.

Intraoperative use of an intensifier is a widely accepted method with which to confirm the femoral anteversion angle; however, a reduction in intraoperative radiation exposure has benefits for both surgeons and patients [35, 36]. We believe that measuring the AW angle may reduce the risk of unnecessary intraoperative radiation overexposure.

The lesser trochanter line is reported as a useful landmark for the prediction of the femoral anteversion angle [24, 25], but it can only be applied when using the posterior approach. In contrast, the regression equation in this study may be applicable not only to the posterior approach but also to other approaches to which the GT is able to be exposed.

This study was performed based on femoral CT models with no deformity of the proximal part of the femur in cases of ONFH and no evidence of deformity or osteophyte formation on the anterior wall of the GT. Therefore, this regression equation may be useful for hip surgery in young patients without deformity, such as transtrochanteric rotational osteotomy which is a joint-preserving surgery for ONFH [29] and internal fixation for femoral neck fracture and slipped capital femoral epiphysis. The anterior wall line of the GT is directly visible, especially in transtrochanteric rotational osteotomy, because the GT is osteotomized, and can help determine the neck osteotomy line to adjust the postoperative femoral anteversion.

The regression equation was not applied for hips showing femoral retroversion because all hips with retroversion showed an AW angle of > 0°. It has been reported that the larger the femoral anteversion angle, the greater the effect of rotation at the femoral neck, and the smaller the femoral anteversion angle, the greater the effect at the femoral shaft [37]. Thus, the femoral retroversion may be regulated at the femoral shaft in the absence of a relationship with the morphology of the anterior wall of the GT. Therefore, the cases with retroversion on plain radiography should be excluded before surgery.

Our study had several limitations. First, the regression equations are not applicable to patients with open epiphyseal growth plates because the study cohort only included patients with closed epiphyseal growth plates. Second, the validation of intraoperative measurement and AW angle in CT is not yet performed. Third, the AW angle was measured at a point 10 mm below the top of the GT. Whether the AW angle is constant in every part of the anterior wall of the GT remains unknown. Fourth, the use of the AW angle to predict the femoral anteversion angle may not be applicable in patients with severe deformity of the proximal femur, including those with end-stage osteoarthritis, osteonecrosis, and Perthes disease. Finally, all data in this study were collected from Japanese people. Previous studies have shown that the femoral anteversion angle does not significantly differ between Japanese males and females of other races, whereas Japanese females have a greater femoral anteversion angle than females of other races [33, 34]. Further study may be necessary before this regression equation is widely adopted, especially for females who are not Japanese.

This study was performed based on CT imaging analysis; thus, for preoperative femoral anteversion prediction, CT examination at least in the proximal part of the femur is necessary. The ideal method with which to predict femoral anteversion would be based on plain radiographs. Further studies will be performed using plain radiography based on the results of this study.

## Conclusion

This CT-based simulation study demonstrated that the femoral anteversion angle can be predicted by the AW angle of the GT. The AW angle may be useful for intraoperative confirmation of the femoral anteversion angle.

## Data Availability

The data sets used and/or analysed during the current study are available from the corresponding author upon reasonable request.

## Abbreviations

CT: Computed tomography
AW: Anterior wall
MRI: Magnetic resonance imaging
GT: Greater trochanter
ONFH: Osteonecrosis of the femoral head
